# Delphi approach to prioritising research in cardiovascular and kidney disease using routinely collected data

**DOI:** 10.1136/bmjopen-2025-113946

**Published:** 2026-04-27

**Authors:** Ross Alexander Forsyth, Amy Coombe, Nigel Brunskill, Timothy Chico, Neeraj Dhaun, Gavin Dreyer, James Fotheringham, Amy Hodgkinson, Jacqueline Ann Langdon MacArthur, Aisling Mcmahon, Eve Miller-Hodges, Mike Molete, Steffen E Petersen, Miranda Scanlon, Anna Stevenson, David C Wheeler, Samira Bell

**Affiliations:** 1 HDR UK, London, UK; 2 British Heart Foundation Data Science Centre, Health Data Research UK, London, UK; 3 Nephrology, University Hospital of Leicester, Leicester, UK; 4 Department of Infection, Immunity and Inflammation, University of Leicester College of Medicine Biological Sciences and Psychology, Leicester, UK; 5 Department of Infection, Immunity and Cardiovascular Disease, The Medical School, The University of Sheffield, Sheffield, UK; 6 University of Edinburgh, Edinburgh, UK; 7 Department of Nephrology, Bart’s Health, London, UK; 8 School of Health and Related Research, University of Sheffield, Sheffield, UK; 9 British Heart Foundation Data Science Centre, Edinburgh, UK; 10 Kidney Research UK, Peterborough, UK; 11 British Heart Foundation Centre for Cardiovascular Science, University of Edinburgh, Edinburgh, UK; 12 Barts Heart Centre, St Bartholomew’s Hospital, London, UK; 13 NIHR Barts Biomedical Research Centre, William Harvey Research Institute, London, UK; 14 Lay Advisory Group, Kidney Research UK, Peterborough, UK; 15 Centre for Nephrology, University College London, London, UK; 16 Division of Population Health and Genomics, University of Dundee, Dundee, UK

**Keywords:** Cardiovascular Disease, Nephrology, Delphi Technique

## Abstract

**Abstract:**

**Objectives:**

Chronic kidney disease (CKD) and cardiovascular disease (CVD) are leading global causes of morbidity and mortality, often coexisting and sharing common risk factors. Despite their interconnection, clinical care and research for affected individuals remain siloed and fragmented. Recognising the need for integrated approaches, this study aimed to identify and prioritise key research questions at the intersection of CKD and CVD that can be addressed using real-world healthcare data to inform more cohesive and data-driven strategies for improving outcomes across both disease areas.

**Design, setting and participants:**

A three-round modified Delphi process was conducted: Round 1 online survey collected open-ended research questions about CKD-CVD priorities via BHF Data Science Centre, Kidney Research UK, UK Renal Health Data Network and HDR UK public involvement channels; Round 2 in-person workshop refined and consolidated items; Round 3 online survey prioritised items across urgency, feasibility and impact using 5-point scales.

**Main outcome measures:**

Survey mean scores for each research question were calculated across the three prioritisation domains, each scored out of 5. The top-ranked questions were identified based on overall scores.

**Results:**

Six thematic domains emerged: risk prediction and early detection, treatment optimisation, health inequities, multimorbidity, disease mechanisms and data infrastructure. The highest-rated research priority was “What are the most effective strategies for prevention, early diagnosis and intervention in CKD?” with a mean score of 12.6 (SD 1.1). Other top priorities included evaluating the cost-effectiveness of early treatment, identifying predictors of kidney failure and assessing the benefits of treating cardiovascular and renal conditions independently.

**Conclusions:**

Across domains, prevention/early detection and early treatment in CKD consistently ranked highest, indicating near-term opportunities for data-enabled cardio-renal research and service improvement; these priorities can inform funder calls, data linkage work and evaluation studies.

**STRENGTHS AND LIMITATIONS OF THIS STUDY**

- A three-round modified Delphi with iterative feedback was performed to develop and refine the questions.
- The panel consisted of a wide range of interest-holders including clinicians, researchers and public contributors.
- There was strong patient and public involvement throughout the process.
- The questions were presented in English; therefore, certain non-English groups may have been excluded.
- There was a potential discipline imbalance in round 3 with more renal researchers which may have influenced the prioritisation.

## Introduction

 Chronic kidney disease (CKD) and cardiovascular disease (CVD) are two of the leading contributors to morbidity and mortality globally, causing suffering to those affected and placing a huge strain on healthcare systems.^1^ Both conditions are highly prevalent and also intricately interconnected bidirectionally through shared risk factors and pathophysiological mechanisms.^2^ CKD is estimated to affect 10–15% of the population worldwide^3 4^ and is strongly associated with adverse cardiovascular outcomes with individuals suffering from CKD six times more likely to die from cardiovascular causes than to progress to end-stage kidney disease.^5^,^7^ In addition, CVDs are well recognised as important risk factors for the development and progression of CKD. The burden of CKD and CVD continues to rise with an ageing population and increasing prevalence of metabolic diseases such as diabetes and obesity.^8 9^ These conditions are associated with reduced quality of life, substantially increased healthcare costs and increased mortality. However, clinical care is often fragmented, limiting the development of integrated prevention and treatment strategies.^10^

The use of real-world healthcare data encompassing patients’ electronic health records, administrative databases, biobanks, registries and patient-reported outcomes provides a transformative opportunity to address these challenges holistically and ultimately improve the lives of patients. Through leverage of large-scale, longitudinal and real-world analyses, big data approaches can uncover novel insights into disease mechanisms, identify high-risk populations and support the evaluation and cost effectiveness of clinical interventions across diverse settings. Platforms such as the NHS England Secure Data Environment (NHSE SDE) enable approved researchers to securely access de-identified patient-level data across multiple care settings, including hospital episodes, emergency care, maternity and mental health.^11^,^13^ However, to fully harness this potential and prioritise resource allocation, there is a critical need to establish a clear and consensus-driven research agenda reflecting the priorities of clinicians, researchers, patients and policymakers. This is particularly important given the increasing burden of multimorbidity and constrained healthcare resource while maintaining high standards of research quality that will deliver the evidence needed to drive effective change.

We therefore conducted a Delphi study involving a multidisciplinary panel of UK experts in nephrology, cardiology, epidemiology, data science and patient advocacy with the aim of identifying and prioritising key research questions that can be addressed using a data-driven approach to improve outcomes for individuals affected by kidney and CVD.

## Methods

**Figure 1:**
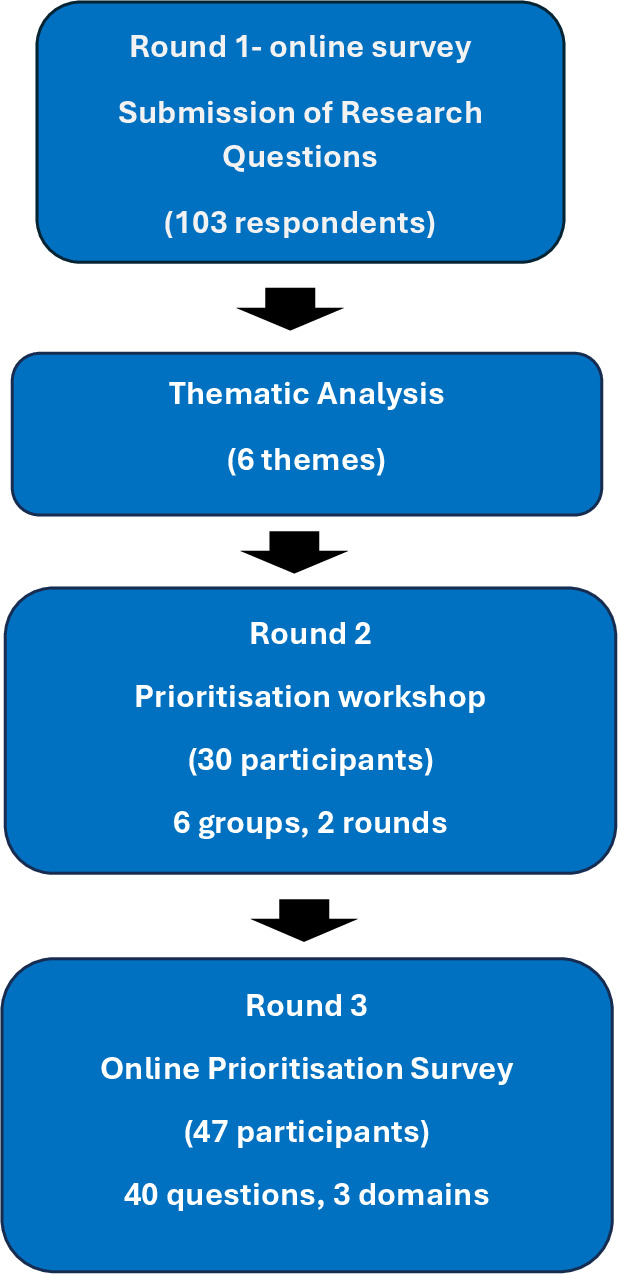
Summary of the three-round Delphi process.

A three-round modified Delphi approach was used to gather and prioritise cardiovascular and renal research questions (figure 1).

The first phase gathered research questions from a diverse range of interest-holders including patients, researchers and healthcare professionals using an online survey. This was followed by a workshop and a further online survey prioritisation round. A steering committee formed of 10 individuals selected on the basis of their expertise by a chair, composed of the study management team from the British Heart Foundation Data Science Centre (BHF DSC), researchers representing renal and CVD and patient/public representatives.

### Prioritisation approach

This modified Delphi focused on priority ranking rather than dichotomous inclusion/exclusion; therefore, we did not set an a-priori percentage agreement. In Round 3, each item was rated on three pre-specified domains (urgency, feasibility using available data and patient/system impact) on a 5-point Likert scale (lowest-highest; ‘unable to rate’ permitted). We calculated domain means and an overall mean (sum of domain means) to rank items.

### Ethical statement

This project was conducted in accordance with the Declaration of Helsinki. This activity was a priority-setting/consensus exercise collecting anonymised opinions from professional and public contributors and did not involve patient health records therefore formal NHS Research Ethics Committee review was not required under UK policy. All participants provided consent before completing each survey and workshop attendees provided consent to participate and for the use of anonymised, non-attributable comments in reporting. No patient identifiable data were collected.

#### Patient and public involvement

We engaged members of the BHF Data Science Centre Public Advisory Group and Kidney Research UK Lay Advisory Group throughout the development, design, management and dissemination phases of this research. The group brought together individuals with a wide range of backgrounds, experiences and interests in data science including those with lived experience of both CKD and CVD.

The first round, which also included patients and the public, was co-designed in collaboration with our public representatives. This collaborative approach ensured the survey met accessibility and plain English requirements. Particular attention was paid to the clarity and inclusivity of all questions and response options. Patients were invited to the second round workshop, with a patient presenting the results of the patient survey.

#### Round 1: online survey

The first round gathered research questions in response to open-ended questions: Question 1: “What are the most important research questions that should be explored using data in relation to cardiovascular and kidney disease?” Question 2: “Please consider how CVD and kidney disease intersect and how data can be used to prevent, manage and treat these conditions.” As described above, alternative questions were posed to patient, public and carers based on feedback from the public contributors: “What challenges in preventing, managing and treating CVD and kidney disease would you most like research to address? How can we use healthcare data better to address these questions?” No limit was placed on the number of responses per individual respondent. These questions were piloted via the BHF DSC Public Advisory group. In order to elicit research questions from a broad and diverse spectrum of interest-holders, the survey was disseminated through multiple channels to maximise reach and engagement. Distribution strategies included targeted invitation emails to members of the UK Renal Health Data Network including patient members, Kidney Research UK and BHF Data Science Centre distribution lists and established patient and public involvement networks (HDR UK Voices,^14^ BHF DSC Patient Advisory Group, Health Data Research UK (HDRUK) Patient Advisory Board and HDRUK Central Patient and Public Involvement and Engagement (PPIE) Team). In addition, there was a dedicated news article on the project website and targeted promotion via social media platforms including LinkedIn, X and YouTube. Responses were submitted through Microsoft forms with no limit to the number of answers per respondent. These responses were categorised into six overarching themes.

#### Round 2: prioritisation workshop

Round 2 of the Delphi process was conducted as a structured in-person workshop involving key interest-holders based on expertise and experience from Round 1. Thirty individuals participated in the workshop. These were a mixture of clinicians/researchers, patient and public contributors and staff from the BHF Data Science Centre and Kidney Research UK. The aim was to refine and prioritise research questions generated during Round 1.

Following analysis of the Round 1 responses (including the patient responses), the questions were categorised into six themes by members of the steering group. Each theme was assigned to a breakout group, with discussions led by a designated member of the steering group to ensure consistency and focus. Each breakout group was carried out twice with each participant attending two breakout groups. Participants were allocated to a group based on their expertise for their first breakout group but were able to choose the second breakout group they attended.

During the workshop, participants engaged in facilitated discussions led by the members of the steering group within their respective thematic groups. The initial questions for each theme based on Round 1 were reviewed by the group, and through collaborative dialogue, refined and additional priorities or gaps were identified. The outcomes of these discussions were summarised by the steering group and revised questions were formulated based on consensus within each group.

#### Round 3: online survey

In Round 3 of the Delphi process, the refined research questions generated during the stakeholder workshop (Round 2) were prioritised through an online survey. This was sent to all those who participated in Rounds 1 and 2. The prioritisation was structured across three domains: urgency, feasibility with available data and potential impact on patients and healthcare systems. These domains were chosen a priori and based on input from the steering group.

Respondents were asked to rate each question within these domains using a five-point ordinal scale: *lowest, low, neutral, high, highest and unable to rate* for each domain (urgency, feasibility and potential impact). The survey was administered via SurveyMonkey and distributed widely to interest-holders through channels employed for Round 1.

#### Statistical analysis

Statistical analyses were conducted using R (version 4.2.1; R Foundation for Statistical Computing, Vienna, Austria) and Microsoft Excel. For each research question, mean scores and standard deviations (mean±SD) were calculated to summarise stakeholder ratings. Questions with the overall highest mean scores across the three domains were considered to represent the highest priorities.

This study is reported in accordance with the ACCORD (ACcurate COnsensus Reporting Document) guideline for biomedical consensus methods, which provides comprehensive standards for reporting Delphi and other consensus studies.^15^

## Results

### Round 1: research questions

**Figure 2:**
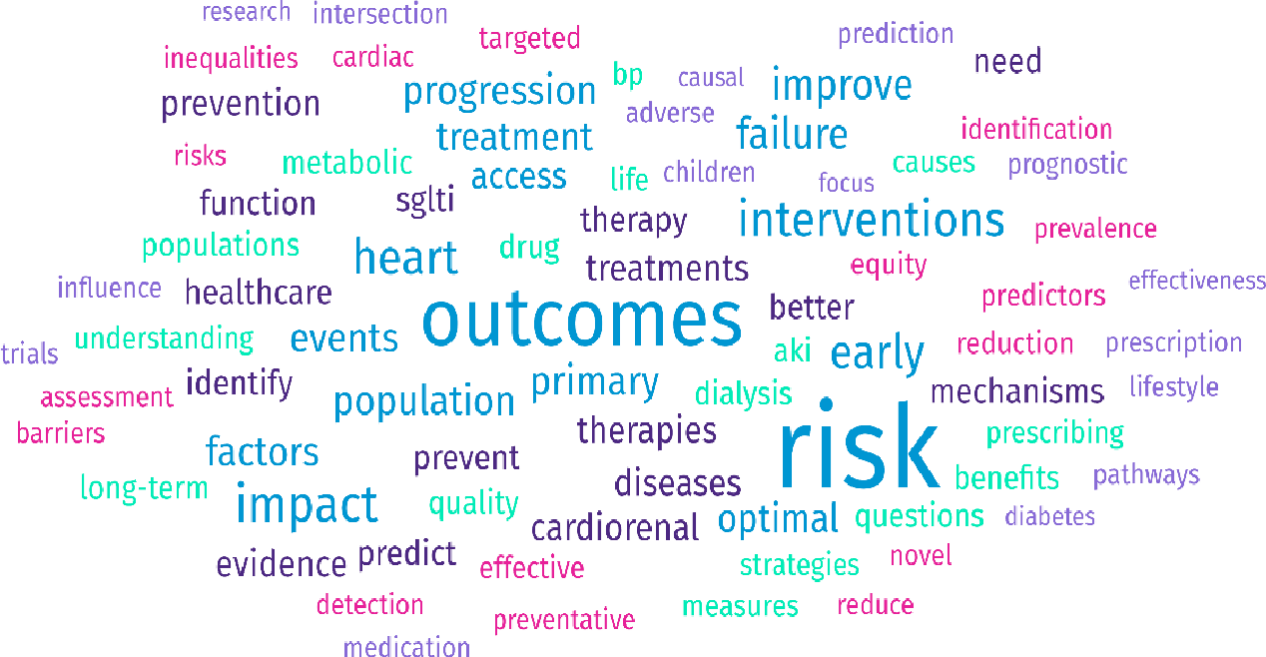
Word cloud of Round 1 responses.

Round 1 was carried out in August 2024. A total of 103 individuals participated in Round 1. Respondents represented a broad range of stakeholder groups, including: clinical academics (n=30), patients, carers or relatives (n=34), NHS professionals (n=10), researchers in kidney disease (n=6), researchers in CVD (n=7), researchers in data science and/or computer science (n=2), charity or patient organisation representatives (n=3), members of the public (n=8) and other roles including student nurses, data scientists and rare disease advocates (n=3). A word cloud displaying the range of topics submitted is shown in figure 2.

There were six dominant thematic domains:

#### Risk prediction and early detection

Respondents prioritised the development of personalised risk prediction tools, including the use of biomarkers, imaging and genetic data. There was strong interest in identifying early indicators of disease progression and using routinely collected data (eg, estimated glomerular filtration rate (eGFR), albuminuria, blood pressure) to stratify individuals at risk and better guide/better direct early intervention.

#### Treatment optimisation and real-world effectiveness

Many responses focused on evaluating the effectiveness and safety of therapies such as sodium-glucose co-transporter-2 inhibitors (SGLT2i), glucagon-like peptide-1 receptor agonists (GLP-1 receptor agonists) and renin-angiotensin-aldosterone system (RAAS) blockers in patients with comorbid kidney and CVD. Respondents also highlighted the need to understand prescribing patterns, barriers to medication optimisation and the role of polypharmacy and multimorbidity.

#### Health inequities and access to care

An important theme was the need to address disparities in diagnosis, treatment and outcomes. Respondents called for research into the impact of sex, ethnicity, socioeconomic status and geography on access to care and health outcomes. Several highlighted the importance of improving equity in referral pathways and treatment provision and uptake.

#### Multimorbidity and complex care pathways

Respondents emphasised the need to understand how cardiovascular, renal and metabolic diseases interact, particularly in patients with multiple long-term conditions. There was interest in integrated care models, shared decision-making and the role of primary care in managing complex patients.

#### Mechanisms and disease interactions

Several responses focused on understanding the biological mechanisms linking kidney and CVD, including inflammation, vascular calcification and the gut microbiome. The impact of acute kidney injury (AKI) on long-term cardiovascular outcomes was also identified as an important issue.

#### Data infrastructure and implementation science

Respondents highlighted the need to improve the quality and linkage of routine health data, including integration of imaging, prescribing and laboratory data. There was strong support for using data to drive quality improvement, evaluate implementation of evidence-based interventions and support pragmatic trials.

### Patient and public priorities

Patient and public contributors emphasised the importance of earlier detection and education around kidney and CVD; better communication of test results and treatment options; research into lifestyle interventions, including diet and exercise and understanding the lived experience of managing both conditions and the need for coordinated care.

### Round 2: workshop

The workshop was held in London on the 5 November 2024. Using the output from the workshop, forty questions were formulated after the combination of questions where possible, removal of duplicates and exclusion of questions that could not be answered using data. These are shown in Box 1. The questions are grouped into six thematic domains, each reflecting a major area of cardio-renal research priority identified by participants:

Box 1 **Full list of questions identified following Round 1**

**Prevention: Risk prediction and stratification**

1. What are the most effective strategies for early diagnosis and intervention in CKD?
2. How can data be used to identify predictors of kidney failure and competing risks in different populations of CKD patients?
3. What are the risk factors for CKD in children?
4. How can early-life interventions reduce the risk of CKD in later life?
5. How can high-risk individuals be identified early in their disease trajectory at key moments (eg, hospital admission with incident AKI, pregnancy)?

**Disease mechanisms and interactions**

1. Can routine healthcare data, including imaging, biomarker and pathology data, be integrated to help identify biological mechanisms underlying the onset and progression of CKD?
2. How do genetic and non-genetic factors contribute to the progression of CKD?
3. How does hypertension influence the progression of CKD?
4. What are the long-term cardiovascular outcomes for patients who have undergone dialysis?

**Health disparities and equity**

1. How does renal and cardiovascular risk vary by GP practice, CCG, health boards and geography?
2. What are the benefits of including diverse patient populations in CKD research?
3. What is the impact of CKD and CVD on severe mental illness and vice versa, and how can interventions targeting mental health improve outcomes for CKD and CVD patients?
4. How can census data be used to study the epidemiology of CKD?
5. What demographic factors are associated with higher CKD prevalence?
6. How do environmental factors like pollution and climate change affect AKI/CKD and CVD in different populations?

**Treatment optimisation and effectiveness**

1. How effective are statins in preventing CKD progression?
2. What proportion of CKD patients are prescribed medication in accordance with NICE guidance?
3. What are the benefits of independently treating cardiovascular and renal elements, such as with SGLT2 inhibitors?
4. Is there a conflict between optimising cardiovascular health and ensuring transplant longevity?
5. What is the impact of cardiac interventions such as transcatheter aortic valve implantation (TAVi) on kidney function?
6. What are the benefits and risks of invasive procedures for individuals with reduced kidney function?
7. How do imaging tests impact the outcomes of invasive procedures in CKD patients?
8. How do discharge medications and compliance affect the long-term cardiovascular health of dialysis patients?

Healthcare utilisation and cost-effectiveness

1. What are the most cost-effective interventions for CKD?
2. What are the costs associated with missed specialist appointments for CKD?
3. What are the benefits and costs of implementing CKD treatments earlier in the disease pathway?
4. What factors influence the variability in cost-effectiveness across different patient groups?
5. How can we maximise the benefits of these treatments while considering cost-effectiveness?
6. What are the economic impacts of CKD on healthcare systems?

**Multi-morbidity and comorbidity**

1. What is the prevalence of musculoskeletal co-morbidities in CKD and CVD patients, and how do they impact on treatment and outcomes?
2. What are the prevalent co-morbidities in a large cohort of CKD and CVD patients?
3. Are there co-morbidities in CKD and CVD patients that are currently under-recognised or unknown?
4. How can cardio-renal disease be defined and understood from data and what existing phenotyping methods are specific to cardio-renal conditions?
5. What are the key differences in disease progression and management between diabetes and CKD?
6. Are there gaps in the management of hypertension and diabetes that affect CKD outcomes?
7. What are the underlying health conditions that link the kidneys and heart?

**Other**

1. How can health apps, wearable devices and point-of-care testing be better integrated into CKD management?
2. How does antibiotic use affect CKD progression?
3. How can patient portals be developed to enhance self-reported health outcomes for CKD patients?
4. What are the potential applications of AI in CKD research and management?

**Prevention, risk prediction and stratification:** questions on early diagnosis, risk factors (including in children), and identifying high-risk individuals at key clinical moments.

**Disease mechanisms and interactions:** questions on biological pathways, genetics, hypertension and long-term cardiovascular outcomes among people with CKD.

**Health disparities and equity:** questions on geographical variation, diverse population inclusion, mental health interactions, demographic drivers of CKD/CVD and environmental influences.

**Treatment optimisation and effectiveness:** questions on medication efficacy, concordance with clinical guidelines, interactions between cardiac and renal treatments and procedure-related risks.

**Healthcare utilisation and cost-effectiveness:** questions on the economic impact of CKD, cost-effectiveness of interventions, variability across patient groups and consequences of missed appointments.

**Multimorbidity and other topics:** questions about comorbidity patterns, phenotyping of cardio-renal disease, hypertension/diabetes management gaps, technology use (apps, wearables), antibiotic effects, patient portals and potential applications of AI.

### Round 3: question prioritisation

**Figure 3:**
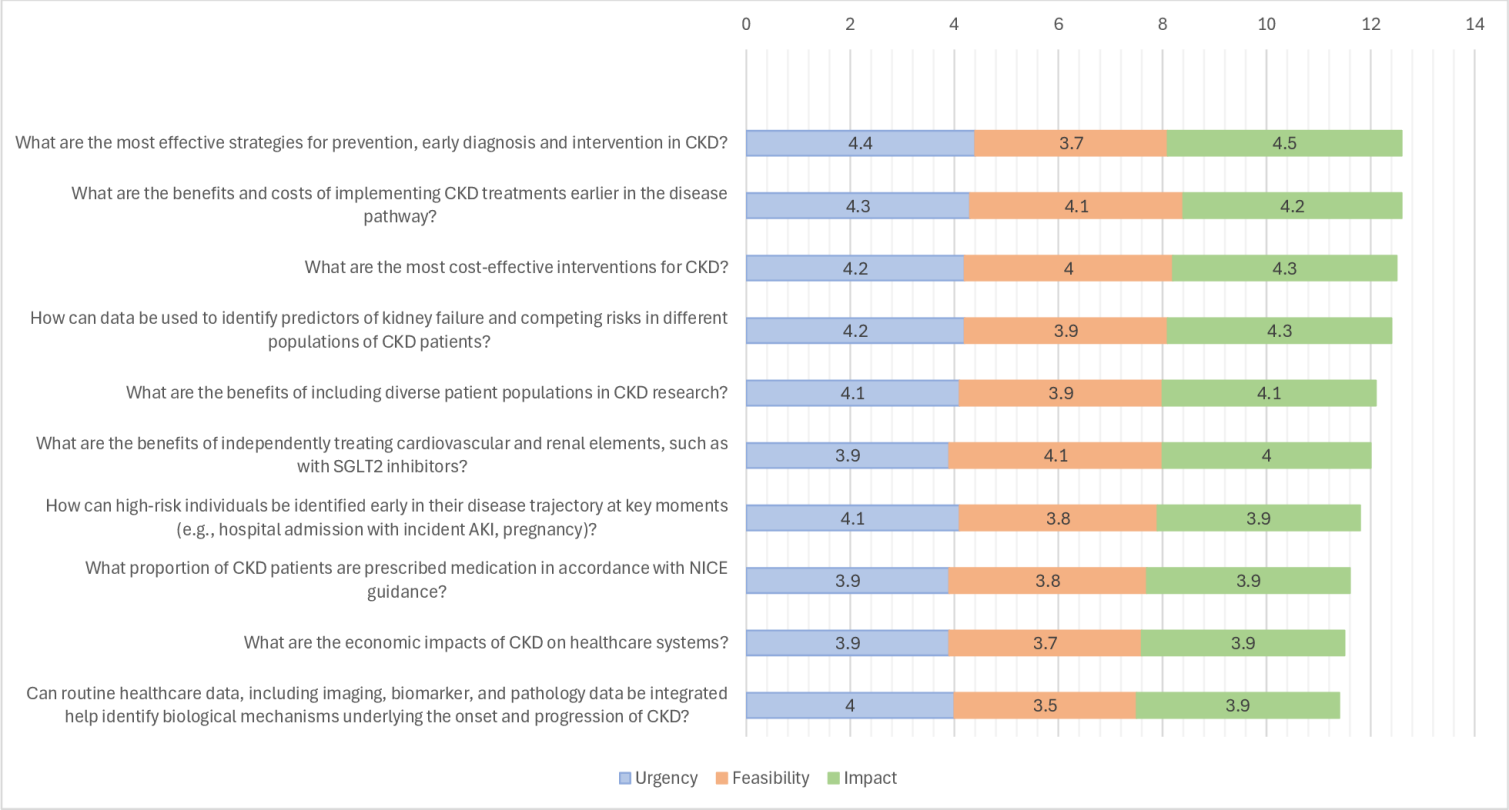
Overall ranking based on three domains (urgency, feasibility and impact) with each domain score out of 5.

The survey was distributed in February 2025. There were 47 participants in this round comprising of including: clinical academics (n=13), patients, carers or relatives (n=6), NHS professionals (n=5), researchers in kidney disease (n=11), researchers in CVD (n=5), researchers in data science and/or computer science (n=2) and members of the public (n=5). Figure 3 summarises the top-ranked questions across all domains, and online supplemental tables 1–3 present the complete set of mean scores for each prioritisation domain.

The highest-rated overall research priority was “What are the most effective strategies for prevention, early diagnosis and intervention in CKD?” (mean 4.4±0.8).

Other highly rated questions included “What are the benefits and costs of implementing CKD treatments earlier in the disease pathway?” (4.3±0.8); “What are the most cost-effective interventions for CKD?” (4.2±0.7); and “How can data be used to identify predictors of kidney failure and competing risks in different populations of CKD patients?” (4.2±0.6).

The question ranked most urgent was “What are the most effective strategies for prevention, early diagnosis and intervention in CKD?” (4.4±0.8); “What are the benefits and costs of implementing CKD treatments earlier in the disease pathway?” (4.3±0.8); and “What are the most cost-effective interventions for CKD?” (4.2±0.8).

The most feasible priorities were “What are the benefits of independently treating cardiovascular and renal elements, such as with SGLT2 inhibitors?” (4.1±0.9); “What are the benefits and costs of implementing CKD treatments earlier in the disease pathway?” (4.1±0.9); and “What are the most cost-effective interventions for CKD?” (4.0±0.9).

The highest impact was attributed to “What are the most effective strategies for prevention, early diagnosis and intervention in CKD?” (4.5±1.0); “How can data be used to identify predictors of kidney failure and competing risks in different populations of CKD patients?” (4.3±0.6); and “What are the most cost-effective interventions for CKD?” (4.3±0.7).

### Participants and panel retention

The composition of the panel for Rounds 1 and 3 is shown in online supplemental table 4. Overall attrition from Round 1 to 3 was 54.4% (103 in Round 1 to 47 in Round 3). The proportion of kidney researchers increased from 5.8% (Round 1) to 23.4% (Round 3), while patients/carers decreased from 33.0% to 12.8%.

## Discussion

We have identified and prioritised key research questions at the intersection of CKD and CVD that can be addressed using real-world data using modified Delphi methodology. Across three iterative rounds involving a diverse group of interest-holders, six thematic domains emerged. These were: risk prediction and early detection, treatment optimisation, health inequities, multimorbidity, disease mechanisms and data infrastructure. The highest-ranked research priority was identifying effective strategies for prevention, early diagnosis and intervention in CKD. Other top priorities included evaluating the cost-effectiveness of early treatment, understanding predictors of kidney failure and assessing the benefits of treating cardiovascular and renal elements independently.

This study engaged a broad spectrum of interest-holders, including clinical academics, NHS professionals, researchers in nephrology and cardiology, data scientists, patients, carers and representatives from patient organisations. Notably, PPI was embedded throughout the process, from survey design to dissemination. This inclusive approach ensured that the proposed research agenda developed reflects both clinical and lived experiences, with PPI contributors highlighting the importance of early detection, education, lifestyle interventions and coordinated care.

Our findings provide a consensus-driven roadmap for data-enabled research in cardio-renal health. Prioritised questions should inform funding calls, guide the development of research proposals and shape national data strategies. The emphasis on prevention, early intervention and health equity aligns with current NHS priorities and underscores the potential of real-world data to support integrated care models leading to patient benefit.

Health economic considerations emerged as a prominent cross-cutting theme in the prioritised research questions. Several of the top-ranked questions explicitly addressed the cost-effectiveness and economic impact of interventions for CKD, reflecting a strong stakeholder interest in ensuring healthcare represented good value. These priorities suggest a clear demand for research that not only improves clinical outcomes but also informs resource allocation, budget planning and policy development. These findings align with current UK health policy directions, including the 2025 NHS 10-Year Health Plan,^16^ which emphasise prevention, early intervention and integrated care for long-term conditions. National strategies such as the UKRI multimorbidity initiative^17^ and HDR UK’s data infrastructure projects further support the use of real-world data to address complex disease interactions.^18^ The emphasis on economic evaluation aligns with the increasing need for financially sustainable healthcare delivery models, particularly in the context of rising multimorbidity and constrained health system capacity. Furthermore, the study highlights the need for improved data infrastructure and linkage to enable robust, population-level analyses that can drive quality improvement and policy change.

This study has several strengths. First, it employed a rigorous, three-round modified Delphi methodology, enabling structured consensus building among a diverse group of interest-holders. This iterative approach ensured that the final research priorities were not only evidence-informed but also reflective of collective expert and public judgement.

Second, the study was grounded in strong PPI. Public contributors were engaged from the outset, co-designing the initial survey and shaping the language and accessibility of the materials. Their continued involvement throughout the process enhanced the relevance and inclusivity of the findings, particularly in highlighting lived experiences and priorities often under-represented in traditional research agendas.

Third, the breadth of stakeholder engagement was a key strength. Participants included clinicians including nephrologists and cardiologists, researchers, patients, carers, data scientists and representatives from charities and public organisations. This multidisciplinary input enriched the prioritisation process and ensured that the identified questions addressed real-world challenges across clinical, research and policy domains.

Finally, our study focused on the use of real-world healthcare data, a rapidly evolving and highly scalable resource. By aligning research priorities with data-driven approaches, the study supports the development of pragmatic, impactful research that can be implemented across healthcare systems.

Several limitations should be noted. Although efforts were made to ensure diverse stakeholder representation, certain groups may have lacked representation. Additionally, as the questions were presented in English, individuals who do not speak English were inadvertently excluded. We did not collect any demographic data on participants and so cannot comment on the diversity of other characteristics beyond role. Moreover, the prioritisation process may have been influenced by the framing of questions or the composition of workshop groups. In addition, scoring of feasibility assessments was based on perceived rather than objective data availability, which may affect the practical implementation of some priorities. Moreover, there were fewer responses in Round 3 which is likely to be due to the higher number of items.^19^ A further limitation was that we were unable to ensure responses were human rather than bots. We purposively sampled the same group of people each round. Free text content affiliation checks did not suggest automation. Finally, renal representation in Round 3 was higher than cardiovascular which could tilt priorities towards CKD centric items.

This Delphi study provides a robust, stakeholder-informed framework for prioritising data-driven research in CKD and CVD. The identified priorities reflect a shared commitment to improving early detection, treatment effectiveness and health equity through the use of real-world data. By aligning research efforts with these priorities, the kidney and cardiovascular research communities can accelerate progress toward better outcomes for patients and more efficient, integrated care systems

## Supplementary material

10.1136/bmjopen-2025-113946 online supplemental file 1

## Data Availability

All data relevant to the study are included in the article or uploaded as supplementary information.
