# Supplementary material for: Delphi approach to prioritising research in cardiovascular and kidney disease using routinely collected data
Source: BMJ Open. 2026 Apr 27;16(4):e113946. doi: 10.1136/bmjopen-2025-113946 (PMC13141130; doi:10.1136/bmjopen-2025-113946)
Supplement: online supplemental file 1 [file bmjopen-16-4-s001.docx]

**Supplementary Material**

**Table 1: Overall Ranking based on 3 domains (urgency, feasibility and impact)**

| **Rank** | **Question** | **Rating**  **(Mean & SD)** |
| --- | --- | --- |
| 1. | What are the most effective strategies for prevention, early diagnosis and intervention in CKD? | 12.6 (1.1) |
| 2. | What are the benefits and costs of implementing CKD treatments earlier in the disease pathway? | 12.5 (0.9) |
| 3. | What are the most cost-effective interventions for CKD? | 12.5 (0.8) |
| 4. | How can data be used to identify predictors of kidney failure and competing risks in different populations of CKD patients? | 12.4 (0.8) |
| 5. | What are the benefits of including diverse patient populations in CKD research? | 12.1 (1.1) |
| 6. | What are the benefits of independently treating cardiovascular and renal elements, such as with SGLT2 inhibitors? | 12.0 (0.8) |
| 7. | How can high-risk individuals be identified early in their disease trajectory at key moments (e.g., hospital admission with incident AKI, pregnancy)? | 11.7 (1.0) |
| 8. | What proportion of CKD patients are prescribed medication in accordance with NICE guidance? | 11.7 (1.1) |
| 9 | What are the economic impacts of CKD on healthcare systems? | 11.6 (0.8) |
| 10. | Can routine healthcare data, including imaging, biomarker, and pathology data be integrated help identify biological mechanisms underlying the onset and progression of CKD? | 11.4 (1.0) |

**Table 2: Questions ranked by Urgency**

| **Rank** | **Question** | **Rating**  **(Mean & SD)** |
| --- | --- | --- |
| 1. | What are the most effective strategies for prevention, early diagnosis and intervention in CKD? | 4.4 (0.8) |
| 2. | What are the benefits and costs of implementing CKD treatments earlier in the disease pathway? | 4.3 (0.8) |
| 3. | What are the most cost-effective interventions for CKD? | 4.2 (0.7) |
| 4. | How can data be used to identify predictors of kidney failure and competing risks in different populations of CKD patients? | 4.2 (0.6) |
| 5. | What are the benefits of including diverse patient populations in CKD research? | 4.1 (1.0) |
| 6. | How can high-risk individuals be identified early in their disease trajectory at key moments (e.g., hospital admission with incident AKI, pregnancy)? | 4.1 (0.8) |
| 7. | Can routine healthcare data, including imaging, biomarker, and pathology data be integrated help identify biological mechanisms underlying the onset and progression of CKD? | 4.0 (0.8) |
| 8. | What proportion of CKD patients are prescribed medication in accordance with NICE guidance? | 3.9 (1.1) |
| 9 | What are the benefits of independently treating cardiovascular and renal elements, such as with SGLT2 inhibitors? | 3.9 (0.8) |
| 10. | What are the economic impacts of CKD on healthcare systems? | 3.9 (0.9) |

**Table 3: Questions ranked by Feasibility**

| **Rank** | **Question** | **Rating**  **(Mean & SD)** |
| --- | --- | --- |
| 1. | What are the benefits of independently treating cardiovascular and renal elements, such as with SGLT2 inhibitors? | 4.1 (0.9) |
| 2. | What are the benefits and costs of implementing CKD treatments earlier in the disease pathway? | 4.1 (0.9) |
| 3. | What are the most cost-effective interventions for CKD? | 4.0 (0.9) |
| 4. | How can data be used to identify predictors of kidney failure and competing risks in different populations of CKD patients? | 3.9 (1.0) |
| 5. | What are the benefits of including diverse patient populations in CKD research? | 3.9 (1.2) |
| 6. | What proportion of CKD patients are prescribed medication in accordance with NICE guidance? | 3.8 (1.1) |
| 7. | How can high-risk individuals be identified early in their disease trajectory at key moments (e.g., hospital admission with incident AKI, pregnancy)? | 3.8 (1.2) |
| 8. | What are the most effective strategies for prevention, early diagnosis and intervention in CKD? | 3.7 (1.2) |
| 9 | What are the economic impacts of CKD on healthcare systems? | 3.7 (1.0) |
| 10. | How does renal and cardiovascular risk vary by GP practice, CCG, health boards, and geography? | 3.7 (1.2) |

**Table 4: Comparison of Panel Composition between Round 1 and 3**

| **Category** | **Round 1 n (%)** | **Round 3 (%)** |
| --- | --- | --- |
| Kidney researchers | 6 (5.8) | 11 (23.0) |
| Patients/carers/relatives | 34 (33.0) | 6 (12.8) |
| Clinical academics | 30 (29.1) | 13 (27.7) |
| NHS professionals | 10 (9.7) | 5 (10.6) |
| Cardiovascular researchers | 7 (6.8) | 5 (10.6) |
| Data science | 2 (1.9) | 2 (4.3) |
| Public | 8 (7.8) | 5 (10.6) |
| Charity/other | 6 (5.8) | 0 (0) |
