# Supplementary material for: Delphi approach to prioritising research in cardiovascular and kidney disease using routinely collected data
Source: BMJ Open. 2026 Apr 27;16(4):e113946. doi: 10.1136/bmjopen-2025-113946 (PMC13141130; doi:10.1136/bmjopen-2025-113946)
Supplement: Supplementary file 2 [file bmjopen-2025-113946.reviewer_comments.pdf]

PEER REVIEW HISTORY

BMJ Open publishes all reviews undertaken for accepted manuscripts. Reviewers are asked to complete a checklist review form and are provided with free text boxes to elaborate on their assessment. These free text comments are reproduced below.

ARTICLE DETAILS

Title (Provisional)

A Delphi Approach to Prioritising Research in Cardiovascular and Kidney Disease Using Routinely Collected Data

Authors

Forsyth, Ross Alexander; Coombe, Amy; Brunskill, Nigel; Chico, Timothy; Dhaun, Neeraj; Dreyer, Gavin; Fotheringham, James; Hodgkinson, Amy; MacArthur, Jacqueline Ann Langdon; Mcmahon, Aisling; Miller-Hodges, Eve; Molete, Mike; Petersen, Steffen E; Scanlon, Miranda; Stevenson, Anna; Wheeler, David; Bell, Samira

---

VERSION 1 - REVIEW

**Reviewer**	**1**
**Name**	**Escalada San Martín , Javier**
**Affiliation**	**Clinica Universitaria de Navarra, Endocrinology and nutrition**
**Date**	**29-Dec-2025**
**COI**	**None**

This is a well-designed, methodologically sound, and clearly reported Delphi study addressing an important gap in cardio-renal research prioritisation. I think it is important to highlight the participation of a multidisciplinary panel of experts in nephrology, cardiology, epidemiology, data science and patient advocacy, although I miss the presence of experts in endocrinology (diabetology).

The results are presented clearly and logically, supported by tables, figures, and supplementary material. The discussion appropriately interprets the findings without overstating their implications. Conclusions are consistent with the data and emphasise relevance to research funding, policy, and integrated care development.

However, in my opinion, the manuscript would benefit from a clearer, explicit statement regarding ethics approval or confirmation that formal approval was not required (as per institutional or national guidance for Delphi studies).

Reviewer 2

Name Okumura, Takahiro

Affiliation Nagoya University Graduate School of Medicine Faculty of Medicine Department of Cardiology, Cardiology

Date 27-Jan-2026

COI None

The authors used a modified Delphi methodology to establish a consensus-driven research agenda for data-enabled cardio-renal research, engaging a multidisciplinary panel of 103 stakeholders across three iterative rounds. These findings are scientifically interesting and timely given the increasing burden of cardio-renal multimorbidity and the expanding availability of linked healthcare datasets. However, this manuscript has some concerns to be solved.

Delphi studies generally prespecify criteria for consensus (e.g., ≥70% agreement or an interquartile range ≤1). In the current manuscript, the consensus definition is not clearly stated. Please specify the criteria used to define and determine consensus, and briefly describe how consensus was reached across rounds.

The attrition rate appears substantial (approximately 54% from Round 1 to Round 3), which may introduce selection bias. Please provide a comparison of participants who completed all rounds versus those who dropped out, particularly with respect to professional background and stakeholder category.

In Round 3, kidney researchers appear to outnumber cardiovascular researchers. This imbalance may have influenced the CKD-oriented nature of the highest-ranked priorities. Please comment on how this distribution may have affected the results and, if possible, provide a sensitivity analysis or stratified summary by stakeholder group.

There is an inconsistency between Figure 1 and Table 1: Figure 1 indicates that 41 questions were included in Round 3, whereas Table 1 lists 40 questions. Please reconcile this discrepancy and revise the figure/table accordingly.

In Figure 3, the question labels are truncated and difficult to read. Please revise the figure to display the full text, or alternatively adopt a numbered labeling system with a corresponding legend listing the complete wording of each item.

Reviewer 3

Name Sanderson, Keia

Affiliation UNC Kidney Center, Nephrology and hypertension

Date 13-Feb-2026

COI **None**

This manuscript describes research priorities in CKM from clinicians, scientists, and community members. Overall this is an important topic and there are many research and implementation gaps and thus a critical need to understand the research priorities from the participants included in this study. Conceptually this was a good idea but the execution and description of the study left me with many more questions that I hope authors can address in a major revision.

Overall Major Concerns

1. I don't see mention of research ethics review, approval, or exemption? This is critical to the scientific community and public trustworthiness.

2. There is some controversary regarding the use of the term "stakeholder." Many articles written about this (one example: Akl EA, et al. "Interest-holders": A new term to replace "stakeholders" in the context of health research and policy. Cochrane Evid Synth Methods. 2024 Oct 29;2(11):e70007. doi: 10.1002/cesm.70007. PMID: 40475280; PMCID: PMC11795957.). Consider revising use of the term throughout.

3. Would use the ACCORD checklist for reporting modified Delphi work to help with clarity around methodology (Logullo P, et al. ACcurate COnsensus Reporting Document (ACCORD) explanation and elaboration: Guidance and examples to support reporting consensus methods. PLoS Med. 2024;21(5):e1004390. PMID: 38709851).

Minor Revisions:

Abstract:

1. Participant recruitment was vague. How many organizations, which public involvement groups?

2. "Round 1 gathered open-ended research questions via an online survey." Again this is vague can you be more specific, for example "open ended research questions about CKD and CVD research priorities."

3. The same applies for how you describe Round 2 and 3 in the abstract. They are quite vague for an abstract that most people will use to decide if they should read your manuscript.

4. The conclusion is underwhelming and vague as well. We know a Delphi study typically provides a consensus-driven result. Would revise to summarize your key point from the study and next steps or future research.

Introduction

1. "Both conditions are not only highly prevalent

but also intricately interconnected bidirectionally through shared risk factors and

pathophysiological mechanisms." Please use direct language, for example "Both conditions are highly prevalent and intricately interconnected...."

2. The second and third paragraphs of the introduction are duplicative of the first paragraph in many places. You start the second paragraph, for example, by saying CVD and CKD are highly prevalent. Yes you just said this. Would greatly revise the second and third paragraphs to reduce duplicative texts.

3. In the beginning you offer to abbreviate CVD for cardiovascular disease but you then inconsistently use it throughout the manuscript starting in the introduction. Would be consistent with CVD abbreviation throughout.

Methods

1. Please define all abbreviations prior to use. For example BHF, PPI, UK, DSC, HDR.

2. Authors describe patient and public involvement participants but also a paragraph is needed to describe healthcare professionals, researchers, and other scientific partners in greater detail.

3. How did you ensure responses were humans and not bots? Was there purposive sampling of the same or different groups of people each round? Were you able to track this at all? If you did not do this please describe it as a limitation.

4. You describe your participants as diverse but there is no demographic data beyond their career or PPI status. Did you collect this, if not please state that demographic data was not collected. If not collected its a limitation.

5. Authors state in the second paragraph of the Methods "The first phase gathered research questions from a diverse range of participants.." but in the "Round 1:Online Survey" paragraph you state "The first round comprise of two more specific open ended questions..." It's a bit confusing because the first description implies more open ended questions were asked. Would revise so that there is congruency between the two paragraphs.

6. How many steering committee members were there? How were they selected. "A steering committee of xx number of persons selected for their expertise or randomly selected or volunteered etc "

7. Round 2 Prioritisation Workshop was the most vague. Again were the participants the same or from a different sampling. Why were they "key?" The description is important to reviewers to understand if and how results may have been biased by conflicts.

8. Following analysis of Round 1 responses, who categorised into six themes? Please describe this, the key participants, the steering committee, the study authors?

9. Each theme was assigned to a breakout group again what was the rationale or organisation there? Random assignment of groups or did all of the nephrologists join one group and cardiologists were in another. The detail is important for contextualizing results.

10. "During the workshop, participants engaged in facilitated discussions within their respective thematic groups." Facilitated discussions led by whom?

11. "The initial questions were reviewed by the group, and through

collaborative dialogue, refined and additional priorities or gaps identified." Which initial questions? The initial questions from Round 1? What were the breakout groups focused on different questions or the same questions?

12. "The outcomes of these discussions were synthesised by the steering group and revised questions were formulated based on consensus..." was this following completion of Round 2? Synthesized how?

13. "In Round 3 of the Delphi process, the refined research questions generated during the stakeholder workshop (Round 2) were prioritised through an online survey." By whom? And to whom was this survey sent to? A different sample of participants? The same participants of the prior rounds?

14. "The prioritisation was structured across three domains: urgency, feasibility with available data, and potential impact on patients and healthcare systems." Please describe why these domains were chosen? They are great domains but were they chosen a priori or based upon the steering group refined questions? Again more detail to help us understand how authors arrived at conclusions.

15. "Respondents were asked to rate each question within these domains using a five-point ordinal scale: lowest, low, neutral, high, highest, and unable to rate." Please be more specific regarding ordinal scale. For example "lowest, low, neutral, high highest" are adjectives of what... interest or priority or etc?

Results

1.Round 2 Workshop including information that really should have been in the methods and Table 1 data should have been summarized instead.

2. " The top ten questions overall and for each prioritisation domain with mean scores are shown in Figure 3 and Supplementary Tables 1-3." This sentence is hard

---

VERSION 1 - AUTHOR RESPONSE

Reviewer: 1
Dr. Javier Escalada San Martín , Clinica Universitaria de Navarra, Carlos III Health Institute, Healthcare Research Institute of Navarra
Comments to the Author:
This is a well-designed, methodologically sound, and clearly reported Delphi study addressing an important gap in cardio-renal research prioritisation. I think it is important to highlight the participation of a multidisciplinary panel of experts in nephrology, cardiology, epidemiology, data science and patient advocacy, although I miss the presence of experts in

endocrinology (diabetology).
Thank you- we have highlighted this and have mentioned that certain groups may have lacked representation.
The results are presented clearly and logically, supported by tables, figures, and supplementary material. The discussion appropriately interprets the findings without overstating their implications. Conclusions are consistent with the data and emphasise relevance to research funding, policy, and integrated care development.
However, in my opinion, the manuscript would benefit from a clearer, explicit statement regarding ethics approval or confirmation that formal approval was not required (as per institutional or national guidance for Delphi studies).

We apologise for this oversight and have now included an ethical statement (see above).

Reviewer: 2
Dr. Takahiro Okumura, Nagoya University Graduate School of Medicine Faculty of Medicine Department of Cardiology
Comments to the Author:
The authors used a modified Delphi methodology to establish a consensus-driven research agenda for data-enabled cardio-renal research, engaging a multidisciplinary panel of 103 stakeholders across three iterative rounds. These findings are scientifically interesting and timely given the increasing burden of cardio-renal multimorbidity and the expanding availability of linked healthcare datasets. However, this manuscript has some concerns to be solved.

Delphi studies generally prespecify criteria for consensus (e.g., ≥70% agreement or an interquartile range ≤1). In the current manuscript, the consensus definition is not clearly stated. Please specify the criteria used to define and determine consensus, and briefly describe how consensus was reached across rounds.
This was a prioritisation exercise and so the situation where an individual would have to agree with one statement (yes/no) did not arise.

We have added:
Prioritisation Approach
This modified Delphi focussed on priority ranking rather than dichotomous inclusion/ exclusion therefore we did not set an a-priori percentage agreement. In Round-3, each item was rated on three pre-specified domains (urgency, feasibility using available data, and patient/system impact) on a 5-point Likert scale (lowest–highest; “unable to rate” permitted). We calculated domain means and an overall mean (sum of domain means) to rank items.

The attrition rate appears substantial (approximately 54% from Round 1 to Round 3), which may introduce selection bias. Please provide a comparison of participants who completed all rounds versus those who dropped out, particularly with respect to professional background and stakeholder category.

We have added a descriptive comparison of Round 1 vs Round 3 participant categories both in the text and supplementary material.
Participants and Panel Retention
The composition of the panel for Rounds 1 and 3 is shown in Supplementary Table 4. Overall attrition from Round 1 to 3 was 54.4% (103 in Round 1 to 47 in Round 3). The proportion of kidney researchers increased from 5.8% (Round 1) to 23.4% (Round3), while patients/carers decreased from 33.0% to 12.8%.

In Round 3, kidney researchers appear to outnumber cardiovascular researchers. This imbalance may have influenced the CKD-oriented nature of the highest-ranked priorities. Please comment on how this distribution may have affected the results and, if possible, provide a sensitivity analysis or stratified summary by stakeholder group.

We have acknowledged this is a limitation in the discussion. Unfortunately, we feel that the low numbers would not allow for meaningful conclusions to be drawn from stratification. Finally, renal representation in Round 3 was higher than cardiovascular which could tilt priorities towards CKD centric items.

There is an inconsistency between Figure 1 and Table 1: Figure 1 indicates that 41 questions were included in Round 3, whereas Table 1 lists 40 questions. Please reconcile this discrepancy and revise the figure/table accordingly.

Apologies- we have now corrected this.

In Figure 3, the question labels are truncated and difficult to read. Please revise the figure to display the full text, or alternatively adopt a numbered labeling system with a corresponding legend listing the complete wording of each item.

We have corrected this.

Reviewer: 3
Dr. Keia Sanderson, UNC Kidney Center
Comments to the Author:
This manuscript describes research priorities in CKM from clinicians, scientists, and community members. Overall this is an important topic and there are many research and implementation gaps and thus a critical need to understand the research priorities from the participants included in this study. Conceptually this was a good idea but the execution and description of the study left me with many more questions that I hope authors can address in a major revision.

Overall Major Concerns
1. I don't see mention of research ethics review, approval, or exemption? This is critical to the

scientific community and public trustworthiness.

Apologies for this oversight. This has now been included (see above).

2. There is some controversary regarding the use of the term "stakeholder." Many articles written about this (one example: Akl EA, et al. "Interest-holders": A new term to replace "stakeholders" in the context of health research and policy. Cochrane Evid Synth Methods. 2024 Oct 29;2(11):e70007. doi: 10.1002/cesm.70007. PMID: 40475280; PMCID: PMC11795957.). Consider revising use of the term throughout.

Thank you- we have revised this throughout.

3. Would use the ACCORD checklist for reporting modified Delphi work to help with clarity around methodology (Logullo P, et al. ACcurate COnsensus Reporting Document (ACCORD) explanation and elaboration: Guidance and examples to support reporting consensus methods. PLoS Med. 2024;21(5):e1004390. PMID: 38709851).

We have included the checklist and a statement within the text.

Minor Revisions:
Abstract:
1. Participant recruitment was vague. How many organizations, which public involvement groups?
2. "Round 1 gathered open-ended research questions via an online survey." Again this is vague can you be more specific, for example "open ended research questions about CKD and CVD research priorities."
3. The same applies for how you describe Round 2 and 3 in the abstract. They are quite vague for an abstract that most people will use to decide if they should read your manuscript.
4. The conclusion is underwhelming and vague as well. We know a Delphi study typically provides a consensus-driven result. Would revise to summarize your key point from the study and next steps or future research.

Thank you for these comments. We have revised the abstract accordingly.
Abstract
Objectives: Chronic kidney disease (CKD) and CVD (CVD) are leading global causes of morbidity and mortality, often coexisting and sharing common risk factors. Despite their interconnection, clinical care and research for affected individuals remains siloed and fragmented. Recognising the need for integrated approaches, this study aimed to identify and prioritise key research questions at the intersection of CKD and CVD that can be addressed using real-world healthcare data to inform more cohesive and data-driven strategies for improving outcomes across both disease areas.
Design, Setting and Participants: A three-round modified Delphi process was conducted: Round-1 online survey collected open-ended research questions about CKD–CVD priorities

via BHF Data Science Centre, Kidney Research UK, UK Renal Health Data Network and HDR UK public involvement channels; Round-2 in-person workshop refined and consolidated items; Round-3 online survey prioritised items across urgency, feasibility, and impact using 5-point scales.
Main Outcome Measures: Survey mean scores for each research question was calculated across the three prioritisation domains each scored out of 5. The top-ranked questions were identified based on overall scores.
Results: Six thematic domains emerged: risk prediction and early detection, treatment optimisation, health inequities, multimorbidity, disease mechanisms, and data infrastructure. The highest-rated research priority was: "What are the most effective strategies for prevention, early diagnosis and intervention in CKD?" with a mean score of 12.6 (SD 1.1). Other top priorities included evaluating the cost-effectiveness of early treatment, identifying predictors of kidney failure, and assessing the benefits of treating cardiovascular and renal conditions independently.
Conclusions: Across domains, prevention/early detection and early treatment in CKD consistently ranked highest, indicating near-term opportunities for data-enabled cardio-renal research and service improvement; these priorities can inform funder calls, data-linkage work, and evaluation studies.

Introduction
1. "Both conditions are not only highly prevalent but also intricately interconnected bidirectionally through shared risk factors and pathophysiological mechanisms." Please use direct language, for example "Both conditions are highly prevalent and intricately interconnected...."

Amended as suggested.

2. The second and third paragraphs of the introduction are duplicative of the first paragraph in many places. You start the second paragraph, for example, by saying CVD and CKD are highly prevalent. Yes you just said this. Would greatly revise the second and third paragraphs to reduce duplicative texts.

Thank you- this has been amended to reduce duplication.

3. In the beginning you offer to abbreviate CVD for cardiovascular disease but you then inconsistently use it throughout the manuscript starting in the introduction. Would be consistent with CVD abbreviation throughout.

We have amended this.

Methods
1. Please define all abbreviations prior to use. For example BHF, PPI, UK, DSC, HDR.

These have been defined.

2. Authors describe patient and public involvement participants but also a paragraph is needed to describe healthcare professionals, researchers, and other scientific partners in greater detail.

This has been added to the results section.

3. How did you ensure responses were humans and not bots? Was there purposive sampling of the same r different groups of people each round? Were you able to track this at all? If you did not do this please describe it as a limitation.

We now acknowledge this as a limitation. Free-text content and affiliation checks did not suggest automation.

4. You describe your participants as diverse but there is no demographic data beyond their career or PPI status. Did you collect this, if not please state that demographic data was not collected. If not collected its a limitation.

This was not collected and now has been acknowledged as a limitation.

5. Authors state in the second paragraph of the Methods "The first phase gathered research questions from a diverse range of participants.." but in the "Round 1:Online Survey" paragraph you state "The first round comprise of two more specific open ended questions..." It's a bit confusing because the first description implies more open ended questions were asked. Would revise so that there is congruency between the two paragraphs.

This has been amended.

6. How many steering committee members were there? How were they selected. "A steering committee of xx number of persons selected for their expertise or randomly selected or volunteered etc "

This information has been added.

7. Round 2 Prioritisation Workshop was the most vague. Again were the participants the same or from a different sampling. Why were they "key?" The description is important to reviewers to understand if and how results may have been biased by conflicts.

Further information has been added.

Round 2: Prioritisation Workshop
Round 2 of the Delphi process was conducted as a structured in-person workshop involving

key interest-holders based on expertise and experience from Round 1. Thirty individuals participated in the workshop. These were a mixture of clinicians/ researchers, patient and public contributors and staff from BHF Data Science Centre and Kidney Research UK . The aim was to refine and prioritise research questions generated during Round 1.
Following analysis of the Round 1 responses (including the patient responses), the questions were categorised into six themes by members of the steering group. Each theme was assigned to a breakout group, with discussions led by a designated member of the steering group to ensure consistency and focus. Each breakout group was carried out twice with each participant attending two different groups. Participants were allocated to a group based on their expertise for their first breakout group but were able to choose the second breakout group they attended.
During the workshop, participants engaged in facilitated discussions led by the members of the steering group within their respective thematic groups. The initial questions for each theme based on Round 1 were reviewed by the group, and through collaborative dialogue, refined and additional priorities or gaps identified. The outcomes of these discussions were summarised by the steering group and revised questions were formulated based on consensus within each group.

8. Following analysis of Round 1 responses, who categorised into six themes? Please describe this, the key participants, the steering committee, the study authors?

This has been included (see above).

9. Each theme was assigned to a breakout group again what was the rationale or organisation there? Random assignment of groups or did all of the nephrologists join one group and cardiologists were in another. The detail is important for contextualizing results.

Further detail has been added (see above).

10. "During the workshop, participants engaged in facilitated discussions within their respective thematic groups." Facilitated discussions led by whom?

Further detail has been added (see above).

11. "The initial questions were reviewed by the group, and through collaborative dialogue, refined and additional priorities or gaps identified." Which initial questions? The initial questions from Round 1? What were the breakout groups focused on different questions or the same questions?

This has been included (see above).

12. "The outcomes of these discussions were synthesised by the steering group and revised questions were formulated based on consensus..." was this following completion of Round 2?

Synthesized how?

This has been clarified (see above).

13. "In Round 3 of the Delphi process, the refined research questions generated during the stakeholder workshop (Round 2) were prioritised through an online survey." By whom? And to whom was this survey sent to? A different sample of participants? The same participants of the prior rounds?

The following text has been added:
This was sent to all those who participated in Round 1 and 2.

14. "The prioritisation was structured across three domains: urgency, feasibility with available data, and potential impact on patients and healthcare systems." Please describe why these domains were chosen? They are great domains but were they chosen a priori or based upon the steering group refined questions? Again more detail to help us understand how authors arrived at conclusions.
The following text has been added:

These domains were chosen a priori and based on input from the steering group.

15. "Respondents were asked to rate each question within these domains using a five-point ordinal scale: lowest, low, neutral, high, highest, and unable to rate." Please be more specific regarding ordinal scale. For example "lowest, low, neutral, high highest" are adjectives of what... interest or priority or etc?

This has been amended:
Respondents were asked to rate each question within these domains using a five-point ordinal scale: lowest, low, neutral, high, highest, and unable to rate for each domain (urgency, feasibility and potential impact)

Results
1.Round 2 Workshop including information that really should have been in the methods and Table 1 data should have been summarized instead.

This has been amended:

Using the output from the workshop, forty questions were formulated after combination of questions where possible, removal of duplicates and exclusion of questions that could not be answered using data. These are shown in Table 1. The questions are grouped into six thematic domains, each reflecting a major area of cardio-renal research priority identified by participants:
Prevention, Risk Prediction & Stratification: questions on early diagnosis, risk factors

(including in children), and identifying high-risk individuals at key clinical moments.
Disease Mechanisms & Interactions questions on biological pathways, genetics, hypertension, and long-term cardiovascular outcomes among people with CKD.
Health Disparities & Equity: questions on geographical variation, diverse population inclusion, mental health interactions, demographic drivers of CKD/CVD, and environmental influences.
Treatment Optimisation & Effectiveness: questions on medication efficacy, concordance with clinical guidelines, interactions between cardiac and renal treatments, and procedure-related risks.
Healthcare Utilisation & Cost-Effectiveness: questions on the economic impact of CKD, cost-effectiveness of interventions, variability across patient groups, and consequences of missed appointments.
Multimorbidity & Other Topics: questions about comorbidity patterns, phenotyping of cardio-renal disease, hypertension/diabetes management gaps, technology use (apps, wearables), antibiotic effects, patient portals, and potential applications of AI.

2. " The top ten questions overall and for each prioritisation domain with mean scores are shown in Figure 3 and Supplementary Tables 1-3." This sentence is hard.

This has been reworded: Figure 3 summarises the top-ranked questions across all domains, and Supplementary Tables 1–3 present the complete set of mean scores for each prioritisation domain.

VERSION 2 - REVIEW

**Reviewer**	**3**
**Name**	**Sanderson, Keia**
**Affiliation**	**UNC Kidney Center, Nephrology and hypertension**
**Date**	**06-Apr-2026**
**COI**	

Thank you for your revisions. Nicely written manuscript. No further recommendations.
